# Predicting infections with multidrug-resistant organisms (MDROs) in neurocritical care patients with hospital-acquired pneumonia (HAP): development of a novel multivariate prediction model

**DOI:** 10.1128/spectrum.02460-24

**Published:** 2025-05-15

**Authors:** Lin Yang, Guangyu Lu, Haiqing Diao, Yang Zhang, Zhiyao Wang, Xiaoguang Liu, Qiang Ma, Hailong Yu, Yuping Li

**Affiliations:** 1 Department of Neurosurgery, Yizheng People’s Hospital, Yizheng, Jiangsu Province, China; 2 School of Public Health, Medical College of Yangzhou University, Yangzhou University 38043, Yangzhou, Jiangsu, China; 3 School of Nursing, Medical College of Yangzhou University, Yangzhou University 38043, Yangzhou, Jiangsu, China; 4 Neuro-Intensive Care Unit, Northern Jiangsu People’s Hospital Affiliated to Yangzhou University, Yangzhou, China; 5 Department of Neurosurgery, Northern Jiangsu People’s Hospital Affiliated to Yangzhou University, Yangzhou, China; Griffith University - Gold Coast Campus, Gold Coast, Queensland, Australia

**Keywords:** neurocritical care patients, MDRO infection, HAP, prediction nomogram, logistic regression, machine learning

## Abstract

Hospital-acquired pneumonia (HAP) is prevalent in the neuro-intensive care unit (NICU), significantly increasing susceptibility to infections with multidrug-resistant organisms (MDROs), which result in high mortality rates and substantial healthcare burdens. Recognition and intervention are paramount. This study aimed to build a prediction model for MDRO infections among NICU patients with HAP. Clinical and laboratory data were collected from the NICU of a grade-A tertiary hospital. Five machine learning models (logistic regression, classification tree, support vector machine, random forest, and K-nearest neighbor) were evaluated based on sensitivity, specificity, accuracy, and receiver operating characteristic curves. A nomogram was developed using the model that performed best in MDRO infection prediction. The performance and clinical utility were assessed using the calibration curve, Brier score, and decision curve analysis. Among 791 neurocritical care patients with HAP, 172 (21.7%) were diagnosed with MDRO infections. The prediction model established by logistic regression exhibited the best performance, with an area under the curve of 0.805. Length of NICU stay (odds ratio [OR] 1.078; 95% confidence interval [CI], 1.070–1.141; *P* < 0.000), number of antibiotics used (OR 1.391; 95% CI, 1.138–1.700; *P* = 0.001), diabetes (OR 1.775; 95% CI, 1.006–3.133; *P* = 0.048), and carbamide (OR 1.038; 95% CI, 1.003–1.074; *P* = 0.035) were significantly correlated with MDRO infections and incorporated into the nomogram. The model demonstrated good calibration (Brier score 0.137). This model can provide clinicians with a tool for prevention and management of MDRO infections in NICU patients with HAP.

**IMPORTANCE:**

Patients with hospital-acquired pneumonia (HAP) in the neuro-intensive care unit (NICU) are at a high risk of developing multidrug-resistant organism (MDRO) infections owing to complex conditions, critical illness, and frequent invasive procedures. However, studies focused on constructing prediction models for assessing the risk of MDRO infections in neurocritically ill patients with HAP are lacking at present. Therefore, this study aims to establish a reliable and easy-to-use nomogram for predicting the risk of MDRO infections in patients with HAP admitted to the NICU. Four easily accessed variables were included in the model, including length of NICU stay, number of antibiotics used, diabetes, and carbamide. This nomogram might help in the prediction and implementation of targeted interventions against infections with MDRO among patients with HAP in the NICU.

## INTRODUCTION

Neurocritical care patients face a substantial risk of nosocomial infections, particularly hospital-acquired pneumonia (HAP), with nearly 40% of patients developing this condition (1–3). Patients with HAP require antibiotic therapy and invasive procedures (4) and are predisposed to developing infections with multidrug-resistant organisms (MDROs) (5, 6). In cases of infections with MDROs, the complexity of treatment and management for patients in neuro-intensive care units (NICUs) with HAP is significantly increased (7), resulting in prolonged hospitalizations, heightened economic burdens for patients and their families, and a heightened risk of in-hospital mortality (8, 9). Therefore, it may be beneficial to utilize machine learning (ML) models for prediction, identification, and application of targeted interventions to reduce the incidence of infections with MDRO among neurocritical care patients with HAP.

The risk factors of infections with MDROs among critically ill patients have been widely studied. For example, the overuse of antibiotics, patient placement in beds in which the prior occupants were infected or colonized with MDROs, and prolonged ICU stays have been identified as important risk factors to the emergence of MDROs among ICU patients (10–14). While several studies have established risk prediction models for infections with MDROs tailored to ICU and NICU settings (12–15), the translation of these approaches into routine clinical practice for risk assessment is very limited (16, 17). This further emphasizes that hospital-level patient information is valuable to assess the risk of a patient suffering infection with MDROs, highlighting the need for the implementation of these methods for MDRO-mediated infection screening in hospitals. Moreover, a risk prediction model for the identification of infections with MDROs for NICU patients with HAP is lacking. Therefore, this study aimed to develop a prediction model for infections with MDROs among neurocritical care patients with HAP using multiple ML methods, including logistic regression, classification tree, support vector machine (SVM), random forest (RF), and K-nearest neighbor (KNN). Moreover, by evaluating and comparing their performance, a visual nomogram was constructed based on the prediction model that performed with the best predictive effect. The ultimate goal was to provide clinical staff with a reliable and easy-to-use tool that can aid in the identification of patients at a higher risk of developing infections with MDROs among NICU patients with HAP.

## MATERIALS AND METHODS

### Study design and participants

A retrospective analysis of patients who were discharged from the NICU was conducted for this study. The inclusion criteria were NICU patients with HAP presenting with an additional infection with at least one MDRO. According to the Centers for Disease Control and Prevention, MDROs are defined as organisms exhibiting simultaneous resistance to three or more classes of antimicrobials commonly used in clinical practice (18). HAP in this study was defined according to the Chinese guidelines for diagnosing and treating HAP and ventilator-associated pneumonia in adults (2018 version) (19), which is consistent with the definition from the American Thoracic Society guidelines (2005 version) (20): clinical symptoms and signs consistent with pneumonia (e.g., fever >38°C, increased white blood cell [WBC] count to >12 × 10^9^, cough, thick sputum with pulmonary rales, and dyspnea) and new, progressive, or persistent pulmonary infiltrates and/or solid lesions on imaging. Non-infected or colonized patients, patients with diseases that severely affected blood chemistry indexes (such as leukemia, severe renal failure, and severe liver failure), and patients with incomplete clinical and laboratory examination data were excluded.

### Antimicrobial susceptibility testing

Matrix-assisted laser desorption/ionization time-of-flight mass spectrometry (bioMérieux, France) was performed for the identification of bacteria. The VITEK-2 Compact (bioMérieux) automatic microbial identification instrument and its accompanying XN04 and N335 cards were used for the determination of drug sensitivity minimum inhibitory concentration values (21). To avoid double counting, only the first isolate was recorded for each patient according to their ID number.

### Sample size calculations

The effective sample size in prediction research (development and validation) was determined by the number of outcome events (22, 23), which was defined to have at least 10 outcome events per variable to ensure accuracy (24). According to existing studies (12, 25, 26), we expected a 20% event rate for infection with MDRO among NICU patients with HAP in this study. To allow five or fewer predictors in the final multivariable logistic regression model, we estimated that 250 patients or more would be required.

### Selection of predictive variables

To comprehensively capture potential predictive variables, we initiated a thorough literature search focusing on indicators for prediction models of infections with MDROs among ICU patients (16, 17). A panel discussion with experts who specialized in critical care was organized to identify variables for inclusion in our analysis.

### Data collection

Patient electronic medical records were retrieved from the hospital’s health information system, and antimicrobial susceptibility test results were acquired from the affiliated microbiology laboratory. We collected the patients’ data, including: (i) demographics (age, sex, time to hospital admission, and Glasgow Coma Scale [GCS] score); (ii) comorbidities (hypertension, diabetes, and coronary heart disease); (iii) laboratory examination findings (levels of albumin, WBCs, serum creatinine, and total protein within 48 h); and (iv) invasive procedures and the use of antibiotics. See Table S1 for more details regarding variable definitions.

### Model development and comparison

The data were randomly divided into a training set (70%) and an internal validation set (30%). Univariate regression analysis was employed in the training set to recognize features. The primary purpose of the training set was to construct models, while the test set was reserved exclusively for assessing the models’ predictive capabilities. Prediction models were established by the application of logistic regression, classification tree, SVM, RF, and KNN. Their performance was evaluated using various metrics, including sensitivity, specificity, accuracy, and receiver operating characteristic curve. A visual nomogram was developed based on the prediction model with the best predictive effect.

Furthermore, a calibration curve and the Brier score were used to evaluate the calibration capacity of the model. The Brier score quantifies the discrepancy between probabilistic predictions and actual outcomes and spans from 0 to 1, with values closer to 0 signifying superior predictions (27). Decision curve analysis (DCA) was performed to assess the clinical utility and practical value of the nomogram. The DCA reflected the net benefit of the model for patients (28).

### Statistical analysis

We first screened for and handled any missing values of variables. When the proportion of missing data was less than 5%, missing values were imputed using the mean filling method. We performed multiple imputation when more than 5% of the data were missing. Furthermore, missing data were treated by casewise deletion if more than 10% of any measure was missing (29).

Continuous data adhering to normal distribution were depicted by mean ± standard deviation, whereas non-normally distributed data were depicted using median values and interquartile ranges. For statistical analysis, the independent-samples *t*-test was employed for continuous variables with normal distribution, and the Mann-Whitney U-test was employed for those with skewed distributions. Categorical variables were represented as percentages or frequencies, and group comparisons were conducted utilizing the chi-squared test. A two-sided *P*-value of under 0.05 was considered as indicative of statistical significance. In order to ensure comprehensive feature selection, we performed univariate regression analysis for feature selection with a significance threshold of *P* < 0.25 for the multivariable logistic regression model. For the other machine learning models, univariate regression analysis was used for feature selection with a significance threshold of *P* < 0.05. Statistical analyses were performed using Stata 15, and the prediction models were evaluated using R version 4.3.1.

## RESULTS

**Fig 1:**
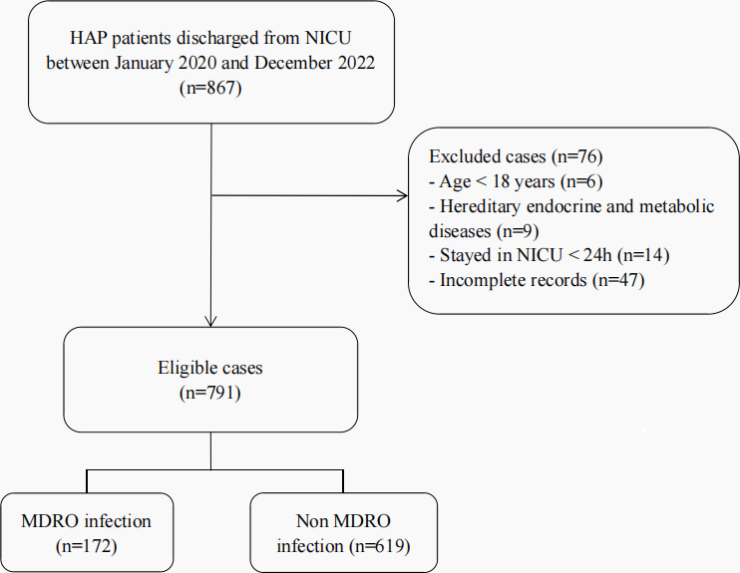
Flow chart for patient inclusion.

This study included 791 NICU patients with HAP. The flow chart for patient inclusion is shown in Fig. 1. Of the 791 patients, 172 (21.7%) were diagnosed with infections with MDROs. Of the 791 NICU patients with HAP, 65.6% were male, with an average age of approximately 60 years (Table 1). Over half of the NICU patients with HAP in the groups with and without infections with MDROs were diagnosed with intracerebral hemorrhage (ICH). Additionally, 56% of the NICU patients with HAP had hypertension (*n* = 443/791), 87.1% required ventilator support (*n* = 689/791), and nearly 97% underwent invasive procedures (*n* = 770/791).

**TABLE 1:** Clinical characteristics of HAP patients from the NICU^*a*^

Feature^*b*^	Total	MDRO group	No MDRO group	*P*-value
	(*n* = 791)	(*n* = 172)	(*n* = 619)	
Demographic characteristics				
Age, year, median (IQR)	62.00 (52.00, 71.00)	64.00 (54.75, 72.00)	61.00 (51.00, 71.00)	0.125
Gender (male), *n* (%)	524 (66.25)	118 (68.6)	406 (65.59)	0.459
BMI, median (IQR)	23.88 (21.60, 26.12)	23.66 (21.43, 25.95)	23.88 (21.64, 26.12)	0.664
GCS, median (IQR)	6.00 (4.00, 10.00)	6.00 (4.00, 10.00)	6.00 (4.00, 10.00)	0.848
Systolic pressure (mmHg), median (IQR)	150.00 (130.00, 174.00)	149.00 (127.00, 175.00)	150.00 (130.00, 174.00)	0.587
Diastolic pressure (mmHg), median (IQR)	86.00 (75.00, 98.00)	85.50 (75.00, 98.25)	86.00 (75.00, 98.00)	0.948
Smoking, *n* (%)	144 (18.2)	32 (18.6)	112 (18.1)	0.967
Drinking, *n* (%)	147 (18.6)	29 (16.9)	118 (19.1)	0.585
Diagnosis				
ICH, *n* (%)	448 (56.6)	97 (56.4)	351 (56.7)	0.942
Traumatic brain injury, *n* (%)	89 (11.3)	9 (5.2)	80 (12.9)	0.005
Arterial aneurysm, *n* (%)	77 (9.7)	18 (10.5)	59 (9.5)	0.715
Infarction, *n* (%)	44 (5.6)	15 (8.7)	29 (4.7)	0.041
Others, *n* (%)	66 (8.34)	18 (10.47)	48 (7.75)	0.123
Comorbidities				
Hypertension, *n* (%)	443 (56.0)	96 (55.8)	347 (56.1)	0.954
Diabetes, *n* (%)	133 (16.8)	38 (22.1)	95 (15.3)	0.036
Infarction, *n* (%)	15 (1.9)	4 (2.3)	11 (1.8)	0.641
Stroke, *n* (%)	79 (10.0)	20 (11.6)	59 (9.5)	0.504
Chronic pulmonary disease, *n* (%)	8 (1.0)	1 (0.6)	7 (1.1)	0.836
Heart disease, *n* (%)	65 (8.2)	17 (9.9)	48 (7.8)	0.458
Cancer, *n* (%)	18 (2.3)	7 (4.1)	11 (1.8)	0.135
Use of ventilator, *n* (%)	689 (87.1)	164 (95.3)	525 (84.8)	<0.001
Days of ventilator use, day, median (IQR)	5.38 (1.94, 9.10)	8.83 (5.00, 13.49)	4.42 (1.29, 8.00)	<0.001
Surgery in admission, *n* (%)	390 (49.3)	103 (59.9)	287 (46.4)	0.002
Invasive procedures				
Deep vein catheterization, *n* (%)	525 (66.4)	120 (69.8)	405 (65.4)	0.287
Indwelling gastric tube, *n* (%)	770 (97.3)	172 (100.0)	598 (96.6)	0.014
Indwelling urethral catheter, *n* (%)	753 (95.2)	165 (95.9)	588 (95.0)	0.758
EVD, *n* (%)	137 (17.3)	41 (23.8)	96 (15.5)	0.015
History of antibiotic use, *n* (%)	740 (93.6)	165 (95.9)	575 (92.9)	0.208
Length of NICU stay, median (IQR)	13.85 (7.00, 24.00)	24.36 (17.00, 31.00)	11.00 (6.00, 20.00)	<0.001
Laboratory indicators				
WBC (10^9^ /L), median (IQR)	11.81 (8.97, 14.93)	11.46 (9.00, 14.33)	11.88 (8.97, 15.04)	0.546
RBC (10^9^ /L), median (IQR)	3.82 (3.27, 4.32)	3.84 (3.23, 4.22)	3.82 (3.28, 4.34)	0.181
Neutrophil ratio (%), median (IQR)	86.40 (81.35, 90.10)	86.10 (81.60, 89.60)	86.70 (81.25, 90.35)	0.371
Lymphocyte ratio (%), median (IQR)	7.50 (4.70, 11.00)	7.60 (4.70, 10.60)	7.50 (4.70, 11.25)	0.991
Neutrophil count (10^9^ /L), median (IQR)	10.05 (7.29, 13.12)	9.81 (7.40, 12.51)	10.16 (7.28, 13.37)	0.475
Lymphocyte count (10^9^ /L), median (IQR)	0.84 (0.56, 1.21)	0.87 (0.54, 1.21)	0.84 (0.57, 1.21)	0.548
Lactic acid (mmol/L), median (IQR)	1.90 (1.20, 2.90)	1.70 (1.20, 2.62)	2.00 (1.30, 3.10)	0.014
Albumin (g/L), median (IQR)	36.39 (31.40, 40.25)	34.60 (30.35, 38.78)	36.70 (32.00, 40.60)	0.004
CRP (mg/dL), median (IQR)	44.62 (11.34, 96.64)	49.83 (10.17, 107.36)	44.36 (12.34, 96.30)	0.896

^*a*^ BMI, body mass index; EVD, external ventricular drainage; RBC, red blood cell; CRP, C-reactive protein.

^*b*^ IQR, Interquartile Range.

Furthermore, the NICU patients with HAP were divided into a training set (553 patients) and a validation set (238 patients), with a ratio of 7:3. In the training set, 122 cases had infections with MDROs (22.1%), and 50 cases in the validation set had infections with MDROs (21%), with no statistical difference between the two sets (Table 2).

**TABLE 2:** Clinical characteristics of patients with HAP in the NICU in the training and validation cohorts^*a*^

Variables	Total	Training set	Testing set	*P*-value
	(*n* = 791)	(*n* = 553)	(*n* = 238)	
MDRO infections (%)	172 (21.7)	122 (22.1)	50 (21.0)	0.814
Demographic characteristics				
Age, year, median (IQR)	62.00 (52.00, 71.00)	63.00 (52.00, 72.00)	59.00 (51.00, 70.00)	0.157
Gender (male), n (%)	524 (66.2)	363 (65.6)	161 (67.6)	0.642
BMI, median (IQR)	23.88 (21.60, 26.12)	24.19 (21.63, 26.13)	23.54 (21.49, 25.95)	0.131
GCS, median (IQR)	6.00 (4.00, 10.00)	7.00 (4.00, 10.00)	6.00 (4.00, 9.00)	0.102
Systolic pressure (mmHg), median (IQR)	150.00 (130.00, 174.00)	150.00 (130.00, 173.00)	152.50 (130.25, 175.75)	0.114
Diastolic pressure (mmHg), median (IQR)	86.00 (75.00, 98.00)	86.00 (75.00, 96.00)	89.00 (76.00, 101.75)	0.033
Smoking, *n* (%)	144 (18.2)	99 (17.9)	45 (18.9)	0.814
Drinking, *n* (%)	147 (18.6)	102 (18.4)	45 (18.9)	0.957
Diagnosis				
ICH, *n* (%)	448 (56.6)	303 (54.8)	145 (60.9)	0.129
Traumatic brain injury, *n* (%)	89 (11.3)	59 (10.7)	30 (12.6)	0.504
Arterial aneurysm, *n* (%)	77 (9.7)	55 (9.9)	22 (9.2)	0.861
Infarction, *n* (%)	44 (5.6)	39 (7.1)	5 (2.1)	0.009
Others, *n* (%)	94 (11.9)	78 (14.2)	16 (6.7)	0.481
Comorbidities				
Hypertension, *n* (%)	443 (56.0)	314 (56.8)	129 (54.2)	0.554
Diabetes, *n* (%)	133 (16.8)	104 (18.8)	29 (12.2)	0.029
Infarction, *n* (%)	15 (1.9)	9 (1.6)	6 (2.5)	0.575
Stroke, *n* (%)	79 (10.0)	53 (9.6)	26 (10.9)	0.655
Chronic pulmonary disease, *n* (%)	8 (1.0)	8 (1.4)	0 (0.0)	0.14
Heart disease, *n* (%)	65 (8.2)	51 (9.2)	14 (5.9)	0.153
Cancer, *n* (%)	18 (2.3)	12 (2.2)	6 (2.5)	0.965
Use of ventilator, *n* (%)	689 (87.1)	541 (86.8)	209 (87.8)	0.064
Days of ventilator use, day, median (IQR)	5.38 (1.94, 9.10)	5.00 (1.75, 8.75)	6.00 (2.00, 10.93)	0.022
Surgery in admission, *n* (%)	390 (49.3)	272 (49.2)	118 (49.6)	0.981
Invasive procedures				
Deep vein catheterization, *n* (%)	525 (66.4)	367 (66.4)	158 (66.4)	1
Indwelling gastric tube, *n* (%)	770 (97.3)	539 (97.5)	231 (97.1)	0.93
Indwelling urethral catheter, *n* (%)	753 (95.2)	531 (96.0)	222 (93.3)	0.14
EVD, *n* (%)	137 (17.3)	84 (15.2)	53 (22.3)	0.021
History of antibiotic use, *n* (%)	740 (93.6)	514 (92.9)	226 (95.0)	0.369
Length of NICU stay, median (IQR)	13.85 (7.00, 24.00)	13.82 (6.97, 23.00)	13.97 (7.00, 25.00)	0.553
Laboratory indicators				
WBC (10^9^ /L), median (IQR)	11.81 (8.97, 14.93)	11.81 (8.97, 14.93)	12.17 (9.59, 15.15)	0.092
RBC (10^9^ /L), median (IQR)	3.82 (3.27, 4.32)	3.80 (3.26, 4.32)	3.87 (3.30, 4.32)	0.433
Neutrophil ratio (%), median (IQR)	86.40 (81.35, 90.10)	86.30 (81.60, 90.00)	86.85 (80.23, 90.62)	0.708
Lymphocyte ratio (%), median (IQR)	7.50 (4.70, 11.00)	7.60 (4.80, 11.00)	7.20 (4.50, 11.23)	0.595
Neutrophil count (10^9^ /L), median (IQR)	10.05 (7.29, 13.12)	9.94 (7.04, 12.85)	10.54 (8.07, 13.66)	0.062
Lymphocyte count (10^9^ /L), median (IQR)	0.84 (0.56, 1.21)	0.83 (0.56, 1.21)	0.86 (0.57, 1.22)	0.669
Lactic acid (mmol/L), median (IQR)	1.90 (1.20, 2.90)	1.90 (1.20, 2.80)	1.92 (1.30, 3.15)	0.182
Albumin (g/L), median (IQR)	36.39 (31.40, 40.25)	36.30 (31.00, 40.20)	36.60 (32.73, 40.32)	0.237
CRP (mg/dL), median (IQR)	44.62 (11.34, 96.64)	43.97 (10.37, 94.70)	52.32 (13.13, 109.83)	0.068

^*a*^ BMI, body mass index; EVD, external ventricular drainage; RBC, red blood cell; CRP, C-reactive protein.

### Distribution of MDROs

A total of 310 strains of MDROs were isolated from 791 NICU patients with HAP, of which 47 patients were infected with three types of MDROs and 49 patients were infected with two types of MDROs. There were 293 strains of gram-negative bacteria, including 100 strains of MDR-*Acinetobacter baumannii* (32.26%), followed by 87 strains of MDR-*Klebsiella pneumoniae* (28.06%), 46 strains of MDR-*Pseudomonas aeruginosa* (14.84%), and 32 strains of MDR-*Escherichia coli* (10.32%). All the cases infected with gram-positive bacteria were methicillin-resistant *Staphylococcus aureus* (MRSA), with a total of 17 strains, accounting for 5.48% of the cases.

### Model development and performance comparison

**Fig 2:**
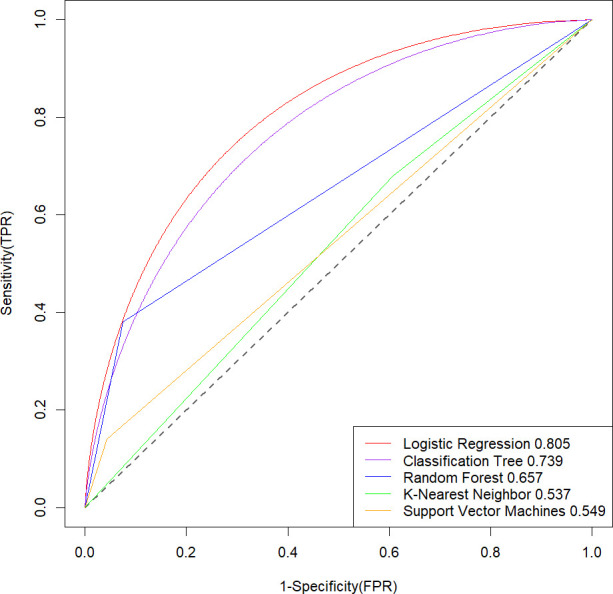
Comparison of the AUC values of the prediction models based on five ML models in predicting infections with MDROs for neurocritical care patients with HAP. TPR (True Positive Rate): The ratio of correctly predicted positive instances to all actual positive samples.FPR (False Positive Rate)**:** The ratio of falsely predicted positive instances to all actual negative samples.

Univariate analysis revealed a total of 20 indicators significantly associated with infections with MDROs (*P* < 0.25), as detailed in Table S2. The percentage of missing data and handling approach are in Table S3. Five models based on logistic regression, classification tree, RF, KNN, and SVM were developed predicting infections with MDROs in NICU patients with HAP. The predictive performances of these models are presented in Fig. 2 and Table 3. For the logistic regression, the optimal solution was found after five major iterations. Table S4 shows the parameter settings of four ML models for predicting infections with MDROs. Among the five models, the logistic regression model had the best predictive effect (AUC: 0.805, 95% CI: 0.7705–0.8526, Sensitivity: 0.963, Specificity: 0.260). Furthermore, the logistic regression model demonstrated the highest accuracy (accuracy: 0.815) among the five models. The multivariate logistic regression analysis showed that four factors were significant predictors of infections with MDROs. As shown, length of NICU stay, number of antibiotics used, diabetes, and carbamide were significantly related to infections with MDROs (Table 4).

**Fig 3:**
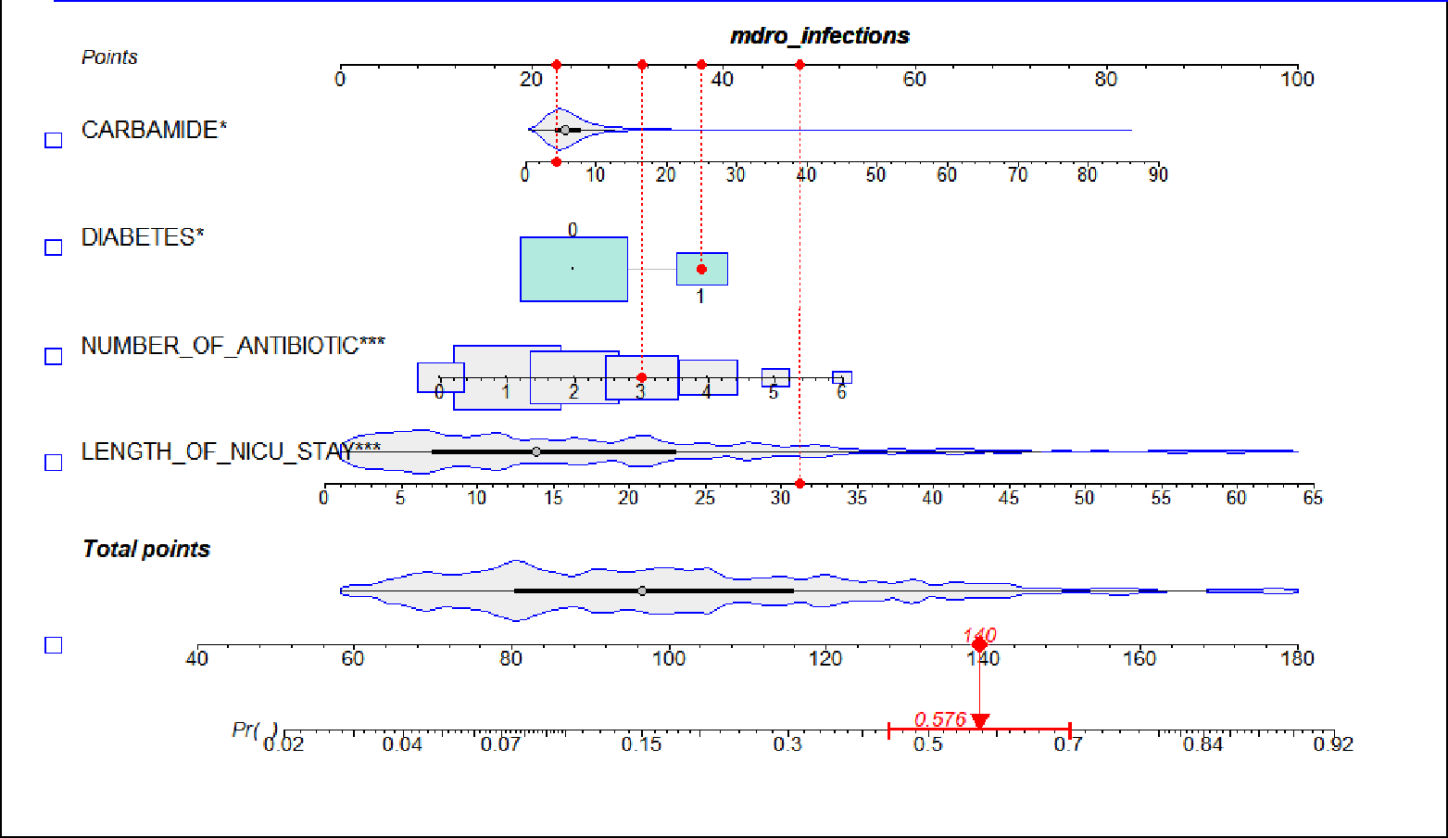
Example of an application of the nomogram to predict infections with MDROs for neurocritical care patients with HAP.

**TABLE 3:** Effectiveness of the five machine learning algorithms

Algorithms	AUC	Sensitivity	Specificity	Accuracy
Logistic regression	0.805	0.963	0.260	0.815
Classification tree	0.739	0.883	0.400	0.781
Random forest	0.657	0.915	0.400	0.807
K-nearest neighbor	0.537	0.394	0.680	0.454
Support vector machines	0.549	0.957	0.140	0.786

**TABLE 4:** Multivariate logistic regression analyses for predicting infections with MDROs among NICU patients with HAP^*a*^

	Multivariate logistic regression analysis		
Variable	Odds ratio	95% CI	*P*-value
Length of NICU stay	1.078	1.055–1.102	<0.001
Gender (male)	0.865	0.564–1.329	0.510
Diabetes	1.775	1.006–3.133	0.048
Heart diseases	1.724	0.808–3.681	0.159
History of surgery	1.287	0.781–2.121	0.323
Ventilator	0.661	0.292–1.498	0.322
Days of ventilator	1.028	0.988–1.070	0.172
EVD	1.280	0.666–2.461	0.459
Lumbar puncture	0.772	0.437–1.364	0.373
Blood transfusion at admission	0.892	0.518–1.535	0.680
Tracheotomy	1.270	0.712–2.266	0.417
Trachea cannula	1.161	0.519–2.599	0.717
Use of antibiotics	0.416	0.135–1.278	0.126
Sedative	1.034	0.516–2.073	0.925
Vaso-active agent	1.487	0.867–2.550	0.149
Glucocorticoid	1.148	0.708–1.862	0.576
Anticoagulant	1.276	0.756–2.155	0.361
Albumin	1.001	0.969–1.036	0.912
Carbamide	1.038	1.003–1.074	0.035
Number of antibiotics	1.391	1.138–1.700	0.001

^*a*^ EVD, external ventricular drainage.

### Nomogram development

Four predictors based on the logistic regression model were integrated into the nomogram. As an example shown in Fig. 3, consider a patient with HAP who had diabetes, received three types of antibiotics during their stay in the NICU, had a carbamide value of 4 mmol/L, and had been in the NICU for 31 days. Based on these factors, the patient’s scores would be approximately 23, 38, 32, and 47, respectively. The sum of these scores, approximately 140, would predict an estimated 57.6% risk of infection with MDRO in this case.

### Clinical application

**Fig 4:**
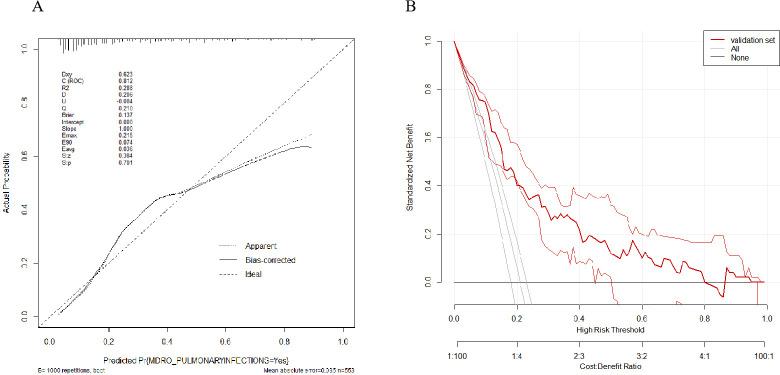
(A) Calibration curve of the nomogram model based on test data. The *y*-axis indicates the actual probability of the patient’s MDRO infections, and the *x*-axis indicates the predicted probability of the patient’s MDRO infections. The 45 degree black dotted line represents the ideal prediction; the solid black line surrounding the 45 degree black dotted line represents the bias-corrected prediction; the black dotted line surrounding the 45 degree black dotted line represents the apparent prediction. (B) Decision curve analysis of the nomogram model. The *y*-axis represents the net benefit. The *x*-axis represents the threshold probability. It shows that if the threshold probability is within a range from 0.01 to 0.80, the use of the nomogram model can bring more net benefit than the patient of complete intervention or no intervention at all.

The calibration curve exhibited good agreement between the nomogram predicting infections with MDROs in patients with HAP and actual observations (Fig. 4A). The Brier score (0.137) underscored the model’s excellent calibration capacity. The DCA for the predictive nomogram for infections with MDROs in NICU patients demonstrated a substantial net benefit across a broad range of threshold probabilities (Fig. 4B).

## DISCUSSION

During the study period, MDROs were identified in 21.7% of NICU patients with HAP. Moreover, in our study, a visualized predictive model was constructed to identify the risk of infections with MDROs for NICU patients with HAP. Compared with other ML-based techniques, the model developed using the logistic regression approach showed a very good predictive effect, revealing that the length of NICU stay, number of antibiotics used, presence of diabetes, and carbamide value were predictors of infections with MDROs in patients with HAP admitted to the NICU.

*Acinetobacter baumannii, Klebsiella pneumoniae*, and *Pseudomonas aeruginosa* demonstrated the top 3 detection rates in our study, aligning with recent research findings within NICU settings (12, 25, 26). However, in contrast to the National Antimicrobial Resistance Surveillance Report released by China in 2022 (which highlighted *Escherichia coli, Klebsiella pneumoniae*, and *Pseudomonas aeruginosa*) (30), *Acinetobacter baumannii* presented the highest detection rates in our analysis. A previous study indicated that MDR-*Acinetobacter baumannii* is being increasingly implicated in infections of critically ill patients (31). *Acinetobacter baumannii* possesses exceptional adherence capabilities, enabling it to colonize in sites such as catheters and ventilators, as well as in the conjunctiva, oral cavity, skin, and respiratory tract of hospitalized patients (32). NICU patients with HAP undergo numerous invasive procedures and have weakened immune systems, rendering them more susceptible to infections caused by MDR-*Acinetobacter baumannii* (33).

The value of carbamide, which has been rarely reported in previous studies, was identified as an independent risk factor for infections with MDRO in our study. Carbamide, the primary end product of amino acid metabolism (34), is linked to kidney function and is also influenced by protein breakdown or intake (35). Carbamide levels may increase in individuals enduring acute infectious diseases, profound fevers, upper gastrointestinal hemorrhage, severe trauma, extensive surgery, and hyperthyroidism and in those on high-protein diets or receiving oral steroid hormone administration (36). Neurocritical care individuals, due to the intense physiological strain coupled with resultant glucocorticoid upregulation, frequently experience elevated rates of protein decomposition, resulting in diminished immune competence and amplified susceptibility to infection (37). Therefore, our findings indicate the importance of maintaining nitrogen balance in patients in the NICU. Timely and effective nutritional therapy is essential for critically ill neurological patients (38). For patients with acute catabolic states and other common pathophysiological features of severe brain injury, nutritional therapy should be initiated as early as possible, preferably within the first 24 h (39). Clinical staff can employ indirect calorimetry to accurately determine the energy requirements of patients, and for those at elevated nutritional risk who are incapable of meeting their requirements orally, early enteral nutrition within 24–48 h after admission is recommended (40).

In our study, diabetes was a significant independent predictor of infections with MDROs among patients with HAP admitted to the NICU. This association can be attributed to the fact that uncontrolled hyperglycemia in patients with diabetes impairs overall immunity through various mechanistic pathways, thereby heightening the risk of infections (41, 42). Clinical staff should regularly monitor and evaluate the blood glucose levels of patients in the NICU with HAP (43). Notably, for neurocritical care patients, strict glycemic control had no effect on patient mortality but increased the incidence of hypoglycemia (44). According to a systematic review and meta-analysis of optimal glycemic control in neurocritical care patients, a more conservative approach is the most appropriate (i.e., maintaining blood glucose levels of 110 mg/dL–180 mg/dL) (45).

The variety of antibiotics used during the hospitalization of patients with HAP in the NICU was strongly associated with infections with MDROs. In NICU settings, patients with HAP commonly experience aspiration and impaired immune function. Moreover, the empirical use of antibiotics, which is routinely advocated for patients with HAP, inevitably leads to increases in both the variety and duration of antibiotic administration (46). Combined use of antibiotics exerts a selective pressure on the patient’s microbial population, fostering the emergence of resistant strains (47). Therefore, stringent adherence to drug indications is paramount (48). Expediting the selection of appropriate antibiotics necessitates the prompt and accurate profiling of antibiotic susceptibility testing (49). Given the approximately 24 h turnaround time for standard antibiotic susceptibility testing following bacterial identification (50, 51), clinicians can turn to ML models to predict antimicrobial resistance in MDROs, which can shorten the delay in obtaining susceptibility testing results from microbiology laboratories (52).

A longer ICU stay was associated with an increased risk of infections with MDROs in the NICU. This finding aligns with previous research indicating that ICU admission itself poses a significant risk for MDRO infections (53, 54). More importantly, patients in the NICU primarily include individuals with traumatic brain injury, spinal cord trauma, acute cerebrovascular disorder, severe central nervous system infection, persistent seizure episodes, and emergent post-neurosurgical care (55). Patients with low immunity are more prone to infections with MDRO. To mitigate this risk of infections with MDROs among NICU patients with HAP, clinical staff should prioritize measures to expedite patient transfer to general wards, as these environments typically exhibit lower levels of bacterial resistance and invasiveness than ICUs do (56). Such strategies can effectively prevent the occurrence of infections with MDROs. However, the lag of length of stay in NICU may limit its clinical application (33).

However, in some studies, male gender and age were included as significant predictors (14, 57, 58), whereas in our analysis, they were not found to be meaningful. This discrepancy may be attributed to the varying male-to-female ratios among patients admitted to different hospitals and departments in different regions. In our study, the proportion of male patients was relatively higher (66.25%). Additionally, patients in the NICU tended to be older, with all 791 included patients being over 51 years of age, which may limit comparability in the analysis. Moreover, C-reactive protein (CRP) has been identified as an important predictor in several studies (14, 59); however, it was not found to be relevant in our research. This could be due to the generally high CRP levels in neurocritical patients (all above 10), with a CRP value greater than 10 indicating acute inflammation. Future studies, particularly multicenter trials with larger sample sizes, are needed to ensure the reliability of these findings.

### Limitations

There are several limitations of our study. First, model development depended on evaluating variables that are routinely collected in primary care and ignored information from the perspective of hospital infection management, such as the implementation rate of hand hygiene and the frequency of pipeline disinfection. Second, this study was limited to a single hospital, potentially limiting the generalizability of our findings. We searched the Medical Information Mart for Intensive Care-IV database (60) but found no data related to infections with MDROs in neurocritical patients with HAP; hence, no external validation was conducted. Third, a degree of internal bias is inevitable in this study due to its nature as a retrospective analysis. To avoid double counting, only the first isolate was recorded for each patient according to their ID number, which may lead to a potential risk of bias. In this retrospective study, some potentially meaningful predictors, such as C-reactive protein, blood urea nitrogen, and whether the patient had experienced aspiration, were not assessed due to the lack of data. The predictor of length of NICU stay in this study exhibits a lag, necessitating future clinical research to explore earlier predictive indicators for identifying high-risk patients.

### Conclusion

In conclusion, the prediction model using the logistic regression method had a better predictive effect than the four other ML methods. Length of NICU stay, number of antibiotics used, diabetes, and the carbamide value were predictors of infections with MDROs for neurocritical care patients with HAP. Based on these predictors, we built a prediction nomogram for the detection of infections with MDROs. For each patient, a higher total point score reflected a greater risk of infection with MDROs. Overall, our study will help in the prediction and implementation of targeted interventions against infections with MDROs among patients with HAP who were admitted to the NICU for better patient management.

## Data Availability

The data analyzed and the codes used during the current study available from the corresponding author on reasonable request.

## SUPPLEMENTAL MATERIAL

Supplemental tables spectrum.02460-24-s0001.docx Tables S1 to S5.

ASM does not own the copyrights to Supplemental Material that may be linked to, or accessed through, an article. The authors have granted ASM a non-exclusive, world-wide license to publish the Supplemental Material files. Please contact the corresponding author directly for reuse.
