# Supplementary material for: Predicting infections with multidrug-resistant organisms (MDROs) in neurocritical care patients with hospital-acquired pneumonia (HAP): development of a novel multivariate prediction model
Source: Microbiol Spectr. 2025 May 15;13(6):e02460-24. doi: 10.1128/spectrum.02460-24 (PMC12131852; doi:10.1128/spectrum.02460-24)
Supplement: Supplemental tables — Tables S1 to S5. [file spectrum.02460-24-s0001.docx]

**Predicting infections with multidrug-resistant organisms (MDROs) in neurocritical care patients with hospital acquired pneumonia (HAP): development of a novel multivariate prediction model**

**Supplementary material**

**Table S1.** The definition of the variables used in this study

| **Variables** | **Variable definition** | **The assignment** |
| --- | --- | --- |
| Age (years) | Age of the patient | Continuous variable |
| Male sex | Patient sex | Female = 0; Male = 1 |
| Length of stay in neuro-ICU (days) | Duration from neuro-ICU admission to discharge | Continuous variable |
| Glasgow score | Glasgow score at admission | Continuous variable |
| Smoking | History of Smoking | No = 0; Yes = 1 |
| Drinking | History of drinking | No = 0; Yes = 1 |
| Diabetes | History of diabetes | No = 0; Yes = 1 |
| Hypertension | History of hypertension | No = 0; Yes = 1 |
| Stroke | History of stroke | No = 0; Yes = 1 |
| Heart disease | History of heart disease | No = 0; Yes = 1 |
| Cerebral infarction | History of cerebral infarction | No = 0; Yes = 1 |
| Chronic obstructive pulmonary disease | History of Chronic obstructive pulmonary disease | No = 0; Yes = 1 |
| Chronic renal insufficiency | History of chronic renal insufficiency | No = 0; Yes = 1 |
| Cancer | History of cancer | No = 0; Yes = 1 |
| Blood transfusion | History of Blood transfusion | No = 0; Yes = 1 |
| Use of ventilator | History of use of ventilator in admission | No = 0; Yes = 1 |
| Days of ventilator | Days of ventilator use in admission | Continuous variable |
| Tracheotomy | History of Tracheotomy in admission | No = 0; Yes = 1 |
| Trachea cannula | History of Trachea cannula in admission | No = 0; Yes = 1 |
| History of Surgery | History of Surgery before admission | No = 0; Yes = 1 |
| Lumbar puncture | History of Lumbar puncture in admission | No = 0; Yes = 1 |
| External ventricular drainage | History of External ventricular drainage in admission | No = 0; Yes = 1 |
| Deep vein catheterization | History of deep vein catheterization in admission | No = 0; Yes = 1 |
| Indwelling gastric tube | History of indwelling gastric tube in admission | No = 0; Yes = 1 |
| Indwelling urethral catheter | History of indwelling urethral catheter in admission | No = 0; Yes = 1 |
| Blood transfusion | Blood transfusion in admission | No = 0; Yes = 1 |
| History of antibiotic use | History of antibiotic use in admission | No = 0; Yes = 1 |
| Sedative | History of Sedative use in admission | No = 0; Yes = 1 |
| Vaso-active agent | History of Vaso-active agent use in admission | No = 0; Yes = 1 |
| KCL | History of KCL use in admission | No = 0; Yes = 1 |
| Glucocorticoid | History of Glucocorticoid use in admission | No = 0; Yes = 1 |
| Anticoagulant | History of Anticoagulant use in admission | No = 0; Yes = 1 |
| Admission diagnosis | Patient's diagnosis was made on admission | Cerebral haemorrhage = 0;  Traumatic brain injury = 1;  Aneurysm = 2;  Infarction = 3;  Brain tumour = 4  Others = 5 |
| BMI, (kg/m^2^) | BMI at admission | Continuous variable |
| LAC | LAC value within 48 hours of admission | Continuous variable |
| Albumin, (g/L) | Albumin value within 48 hours of admission | Continuous variable |
| Carbamide, (mmol/L) | Carbamid value within 48 hours of admission | Continuous variable |
| Total protein, (g/L) | Total protein value within 48 hours of admission | Continuous variable |
| WBC, (10^9/L) | WBC value within 48 hours of admission | Continuous variable |
| Hemoglobin, (g/L) | Hemoglobin value within 48 hours of admission | Continuous variable |
| CRP (mg/dl) | CPR value within 48 hours of admission | Continuous variable |
| Neutrophil ratio (%) | Neutrophil ratio within 48 hours of admission | Continuous variable |
| Lymphocyte ratio (%) | Lymphocyte ratio within 48 hours of admission | Continuous variable |
| Neutrophil count (10^9/L) | Neutrophil count within 48 hours of admission | Continuous variable |
| Lymphocyte count (10^9/L) | Lymphocyte count within 48 hours of admission | Continuous variable |
| Procalcitonin, (ng/ml) | Procalcitonin within 48 hours of admission | Continuous variable |
| ALT, (U/L) | ALT within 48 hours of admission | Continuous variable |
| AST, (U/L) | AST within 48 hours of admission | Continuous variable |
| Uric Acid, (umol/L) | Uric Acid within 48 hours of admission | Continuous variable |
| Glucose, (mmol/L) | Glucose within 48 hours of admission | Continuous variable |
| Number of antibiotics* | Number of antibiotic used by patients prior to the onset of CRKP infection | Continuous variable |

Note:INR,International Normalized Ratio; BMI: Body mass index; RBC, red blood cell; WBC, white blood cell; ALT, Alanine transaminase; AST, Aspartate transaminase; EVD: External Ventricular Drainage;LAC, Lactic acid.

**Table S2.** Univariate analyses for predicting MDROs infection in NICU patients with HAP.

| **Variables** | **Z** | **P value** |
| --- | --- | --- |
| Age (years) | 0.45 | 0.655 |
| Male sex | 1.66 | 0.096 |
| Length of stay in neuro-ICU (days) | 8.39 | 0.000 |
| Glasgow score | -0.64 | 0.520 |
| Smoking | -0.76 | 0.448 |
| Drinking | -0.92 | 0.355 |
| Diabetes | 2.10 | 0.036 |
| Hypertension | 0.56 | 0.573 |
| Stroke | 0.80 | 0.423 |
| Heart disease | 1.67 | 0.095 |
| Cerebral infarction | 0.01 | 0.991 |
| Chronic obstructive pulmonary disease | -0.64 | 0.519 |
| Chronic renal insufficiency | 0.15 | 0.882 |
| Cancer | 0.94 | 0.348 |
| Blood transfusion | 0.43 | 0.664 |
| Use of ventilator | 2.02 | 0.044 |
| Days of ventilator | 5.29 | 0.000 |
| Tracheotomy | 3.58 | 0.000 |
| Trachea cannula | 2.23 | 0.026 |
| History of Surgery | 1.75 | 0.080 |
| Lumbar puncture | 2.15 | 0.032 |
| External ventricular drainage | 2.12 | 0.034 |
| Deep vein catheterization | 0.22 | 0.822 |
| Indwelling urethral catheter | -0.08 | 0.939 |
| Blood transfusion | 2.6 | 0.009 |
| History of antibiotic use | 1.42 | 0.156 |
| Sedative | 1.29 | 0.198 |
| Vaso-active agent | 1.95 | 0.051 |
| KCL | 0.07 | 0.945 |
| Glucocorticoid | 2.11 | 0.035 |
| Anticoagulant | 3.17 | 0.002 |
| BMI, (kg/m^2^) | -0.58 | 0.562 |
| LAC | -0.90 | 0.368 |
| Albumin, (g/L) | -1.99 | 0.047 |
| Carbamide, (mmol/L) | 1.58 | 0.115 |
| Total protein, (g/L) | -0.49 | 0.621 |
| WBC, (10^9/L) | 1.09 | 0.274 |
| Hemoglobin, (g/L) | 1.10 | 0.270 |
| CRP (mg/dl) | 0.25 | 0.804 |
| Neutrophil ratio (%) | -0.98 | 0.329 |
| Lymphocyte ratio (%) | -0.83 | 0.407 |
| Neutrophil count (10^9/L) | -0.42 | 0.675 |
| Lymphocyte count (10^9/L) | -0.69 | 0.490 |
| Procalcitonin, (ng/ml) | 0.97 | 0.331 |
| ALT, (U/L) | -0.15 | 0.884 |
| AST, (U/L) | 0.18 | 0.855 |
| Uric Acid, (umol/L) | 0.57 | 0.569 |
| Glucose, (mmol/L) | 1.12 | 0.263 |
| Number of antibiotics* | 6.73 | 0.000 |

Note:INR,International Normalized Ratio; BMI: Body mass index; RBC, red blood cell; WBC, white blood cell; ALT, Alanine transaminase; AST, Aspartate transaminase; EVD: External Ventricular Drainage;LAC, Lactic acid.

**Table S3.** Percentage and handling of missing data

| **Variables** | **Percentage of missing data (%)** | **Handle approach** |
| --- | --- | --- |
| Age (years) | 0 |  |
| Male sex | 0 |  |
| Length of stay in neuro-ICU (days) | 0 |  |
| Glasgow score | 3 | mean filling |
| Smoking | 0 |  |
| Drinking | 0 |  |
| Diabetes | 0 |  |
| Hypertension | 0 |  |
| Stroke | 0 |  |
| Heart disease | 0 |  |
| Cerebral infarction | 0 |  |
| Chronic obstructive pulmonary disease | 0 |  |
| Chronic renal insufficiency | 0 |  |
| Cancer | 0 |  |
| Blood transfusion | 0 |  |
| Use of ventilator | 0 |  |
| Days of ventilator | 0 |  |
| Tracheotomy | 0 |  |
| Trachea cannula | 0 |  |
| History of Surgery | 0 |  |
| Lumbar puncture | 0 |  |
| External ventricular drainage | 0 |  |
| Deep vein catheterization | 0 |  |
| Indwelling gastric tube | 0 |  |
| Indwelling urethral catheter | 0 |  |
| Blood transfusion | 0 |  |
| History of antibiotic use | 0 |  |
| Sedative | 0 |  |
| Vaso-active agent | 0 |  |
| KCL | 0 |  |
| Glucocorticoid | 0 |  |
| Anticoagulant | 0 |  |
| Admission diagnosis | 0 |  |
| BMI, (kg/m^2^) | 4 | mean filling |
| LAC | 9 | multiple imputation |
| Albumin, (g/L) | 7 | multiple imputation |
| Carbamide, (mmol/L) | 6 | multiple imputation |
| Total protein, (g/L) | 4 | mean filling |
| WBC, (10^9/L) | 7 | multiple imputation |
| Hemoglobin, (g/L) | 4 | mean filling |
| CRP (mg/dl) | 9 | multiple imputation |
| Neutrophil ratio (%) | 3 | mean filling |
| Lymphocyte ratio (%) | 3 | mean filling |
| Neutrophil count (10^9/L) | 4 | mean filling |
| Lymphocyte count (10^9/L) | 4 | mean filling |
| Procalcitonin, (ng/ml) | 9 | multiple imputation |
| ALT, (U/L) | 7 | multiple imputation |
| AST, (U/L) | 7 | multiple imputation |
| Uric Acid, (umol/L) | 6 | multiple imputation |
| Glucose, (mmol/L) | 7 | multiple imputation |
| Number of antibiotics* | 0 |  |
| hypersensitive C-reactive protein | 43 | Delete |
| platelet distribution width | 31 | Delete |
| mean platelet volume | 23 | Delete |
| OSM | 37 | Delete |

**Table S4** Parameters settings of four ML models

| Model | Parameters settings |
| --- | --- |
| Classification Tree | Default parameter：minsplit = 20; minbucket = 7 |
| Random Forest | ntree=5000 |
| K-Nearest Neighbor | k = 10; distance = 1 |
| Support Vector Machines | kernel = "sigmoid"; gamma = c(0.1, 0.5, 1, 2, 3, 4); coef0 = c(0.1, 0.5, 1, 2, 3, 4) |

**Table S5** Results of resampling the train and test sets

| **Algorithms** | **AUC** | **Sensitivity** | **Specificity** | **Accuracy** | **AIC** |
| --- | --- | --- | --- | --- | --- |
| Logistic Regression | 0.804 | 0.931 | 0.408 | 0.823 | 477.1 |


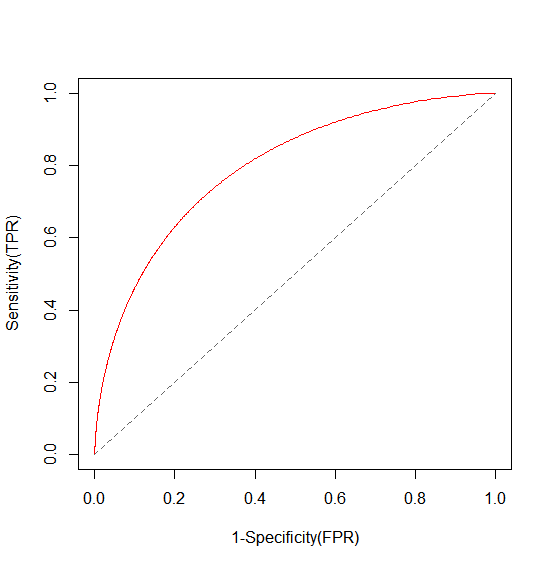


**Figure S1.** ROC curve of resampling the train and test sets


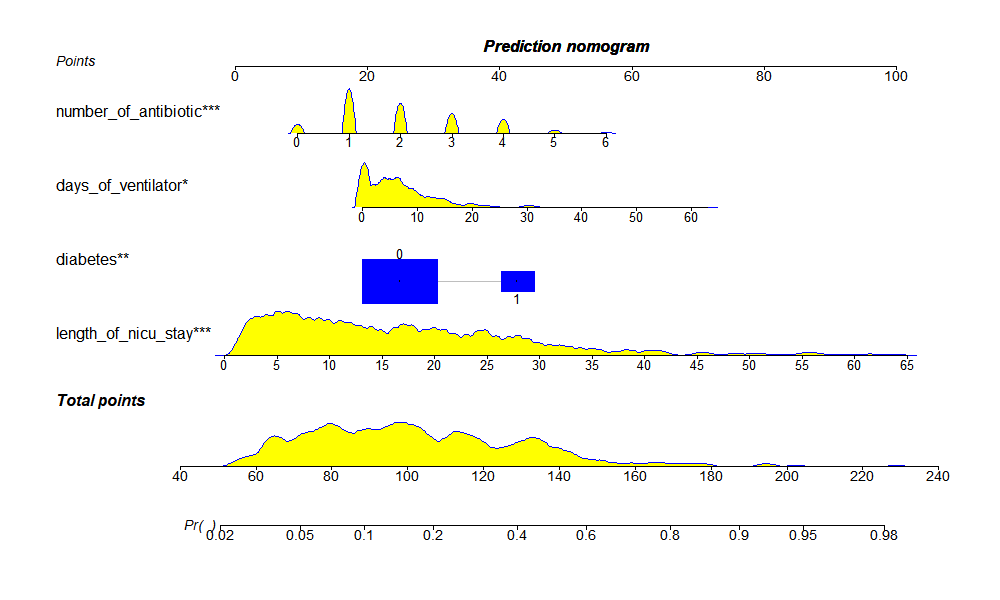


**Figure S2**. Nomogram of resampling the train and test sets
